# The impact of complementary feeding foods of animal origin on growth and the risk of overweight in infants

**DOI:** 10.1093/af/vfz037

**Published:** 2019-09-28

**Authors:** Minghua Tang

**Affiliations:** Section of Nutrition, Department of Pediatrics, University of Colorado School of Medicine, University of Colorado Anschutz Medical Campus, Aurora, CO

**Keywords:** animal protein, growth, microbiome

**Implications**

- Evidence-based dietary recommendations during early complementary feeding are lacking. Despite extensive research of protein intake in infant formula on growth and risk of overweight, research focusing on the impact of protein intake during early complementary feeding is needed.
- Current literature, although limited, showed a potential effect of meat intake to promote infant linear growth (length gain), which in turn led to a decreased risk of overweight in some studies.
- Possible mechanisms regarding protein intake and infant growth are not clear and the early protein hypothesis cannot explain the differential effect of various sources of protein on infant growth during early complementary feeding. Emerging animal studies showed that the gut microbiome may mediate the dietary impact on infant growth.

## Introduction

Evidence-based consensus holds that the first year of life is critical in obesity programming and unfavorable infant growth patterns, namely, excessive weight gain in relation to length gain or increased weight-for-length *Z* score, is strongly associated with obesity in young children and adolescents. Given the current obesity rates in U.S. children, identifying modifiable risk factors underpinning excessive weight and adiposity gain early in life are urgently needed. Although extensive research has been done on infant formula consumption and risk of overweight, a significant knowledge gap exists in the effects of complementary feeding on growth and risk of overweight during late infancy, especially regarding protein-rich foods. This review will present current literature on the impact of complementary foods of animal origin on growth trajectory and the risk of overweight in infants and discuss the potential mechanisms linking protein-rich complementary foods to infant growth and future research recommendations.

## Protein Intake in Infant Formula and the Risk of Overweight

It was first reported by Rolland et al. (Rolland-Cachera *et al.*, 1995) that protein intake at 2 yr of age, not carbohydrate or fat, was positively associated with body mass index at 8 yr of age. The DARLING study (Dewey *et al.*, 1992) was one of the first to demonstrate that formula-fed infants had a greater weight-for-length score compared with breastfed infants. In this study, breastfed and formula-fed infants were followed from birth to 18 mo of age. Findings from this study showed that the mean weight-for-length score, the parameter of risk of overweight from 0 to 24 mo, became significantly higher in formula-fed infants starting at 6 mo and continued until the end of the observation. Because standard infant formula has a 46% higher protein than breastmilk (~2.2 vs. ~1.5 g protein/100 kcal of formula), this discrepancy has been considered the key contributor to greater weight-for-length score and increased risk of overweight in formula-fed infants. Indeed, a large-scale randomized controlled trial conducted in Europe (Koletzko *et al.*, 2009) compared iso-caloric infant formula with high- and low-protein from birth to 12 mo. Results showed that the high-protein formula led to more rapid weight gain, adiposity, and a higher weight-for-length score compared with the low-protein formula, despite same energy intakes. These differences were also persistent at school age (Totzauer *et al.*, 2018).

Current consensus holds that the high protein content in formula contributes to early rapid weight gain and increased risk of overweight in infants. The current recommendation proposed by the European Society for Pediatric Gastroenterology, Hepatology, and Nutrition is to limit protein during infancy to ≤15% of energy, without clear distinction for sources. Although the premise of this recommendation was based primarily on infant formula studies, the recommendation concerns both liquid diet and complementary feeding phases. A significant knowledge gap exists in the effects of common types of protein-rich foods on growth during early complementary feeding. As in dietary recommendations for adults, recommendations during complementary feeding need to distinguish between types of protein-rich foods, because dairy is no longer the sole protein source available.

## Importance of Complementary Feeding

**Figure 1.**
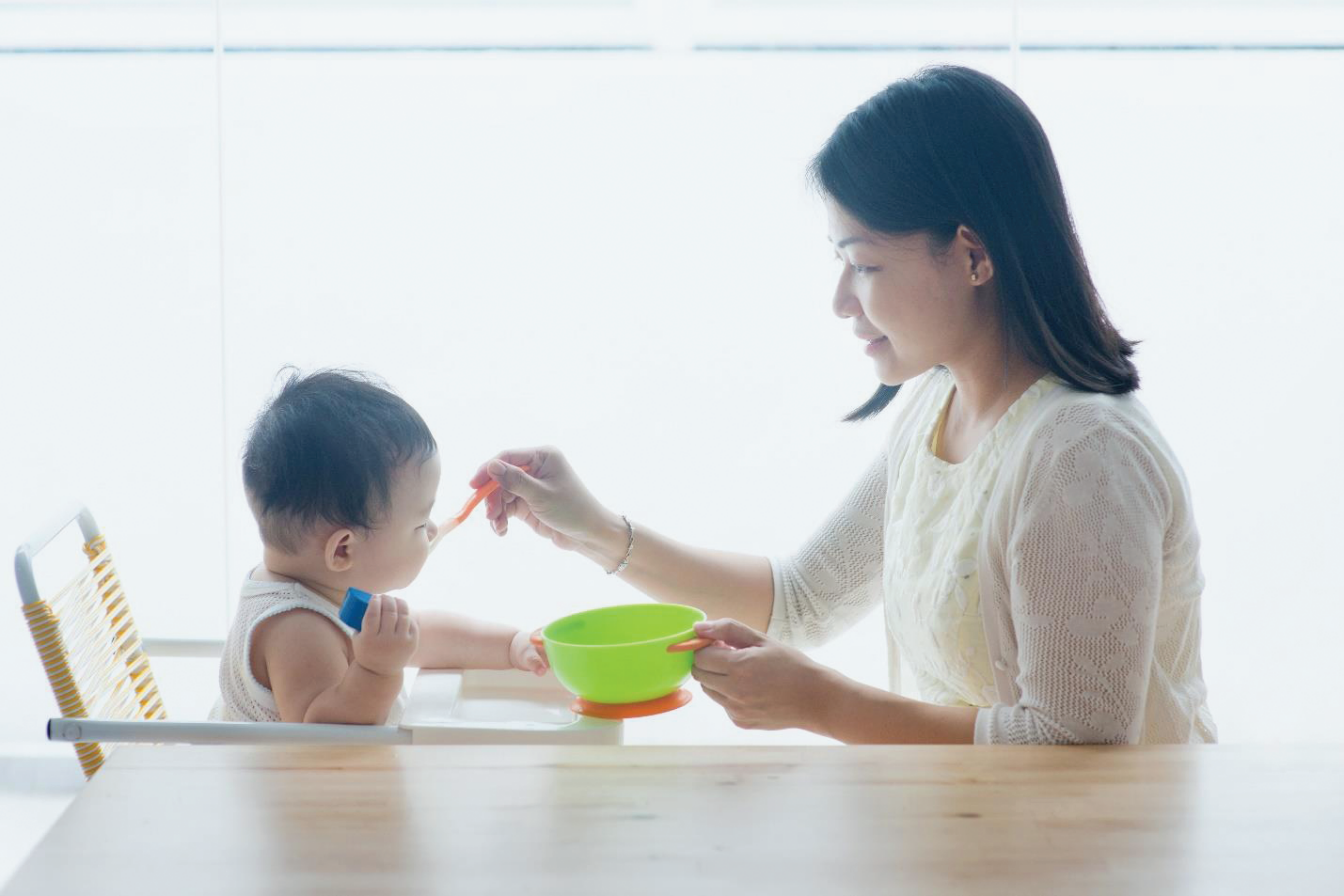
Complementary feeding begins around 5 to 12 mo of age and represents the time when solid foods are progressively introduced to infants who no longer rely solely on breastmilk or formula.

Early complementary feeding (~5 to 12 mo) represents the time when solid foods are progressively introduced to infants who no longer rely solely on breastmilk or formula (Figure 1). It is a unique and malleable period to establish lifelong dietary patterns and food preference, and a window of opportunity to develop interventions to prevent risk of overweight and obesity later in life. Different from toddlerhood (13 to 24 mo) when the individual is fully exposed to table foods and family meals, early complementary feeding has less food diversity, which makes it challenging and yet critical to provide evidence-based recommendations to meet nutrient needs while preventing undesired growth patterns. However, current complementary feeding recommendations are largely driven by tradition and marketing rather than a sound evidence base. Both the National Institute of Health and United States Department of Agriculture recognized the pressing knowledge gaps and emphasized the need for evidence-based dietary guidance during complementary feeding. The current Dietary Guidelines for Americans have minimal guidance for infants because of insufficient research available. The B-24 (birth to 24 mo) project was thus initiated to evaluate evidence and encourage research in high priority topics. In particular, “differences in protein intake, total amount and source” on growth, overweight, and obesity is listed as the first research priority topic for older infants (6 to 12 mo of age).

## Consumption of Animal-Based Complementary Foods

Current national surveys indicate an overall low protein intake from animal origins in older infants. The Feeding Infants and Toddlers Study (FITS) is the largest dietary intake survey of infants and toddlers in the United States. FITS 2008 showed that less than 10% of older infants consumed meat which accounted for <5% of total energy (Reidy *et al.*, 2017). The recently published FITS 2016 data also showed <5% of older U.S. infants consumed baby-food meats (Bailey *et al.*, 2018) and similar patterns were found in Canadian infants (Friel *et al.*, 2009). Specifically, in 4- to 6-mo-old U.S. infants, baby-food meats were the least consumed complementary foods while infant cereal was the most commonly consumed. In 6- to 12-mo-old infants, the exposure to meat and dairy foods increased to 41% of participants surveyed (one single 24-h recall) and the mean total protein intake was 9.2% of total calories (Bailey *et al.*, 2018). Likewise, National Health and Nutrition Examination Survey 2009 to 2012 data on nutrient intakes also showed that the average total protein intake in 6- to 11-mo-old infants were 10% or 2 g/kg/d, without specifying protein source. Overall, older U.S. infants, on average, do not consume meat as the primary protein source of complementary foods and the low meat intake may at least partially contribute to the increased percentage of infants falling short on recommended iron intake (18% in 2016).

Effects of animal-based protein-rich foods during complementary feeding on infant growth and risk of overweight have not been thoroughly studied and available randomized controlled trials were primarily focused on meat (Engelmann *et al.*, 1998; Makrides *et al.*, 1998; Yeung and Zlotkin, 2000; Krebs *et al.*, 2006; Dube *et al.*, 2010; Tang *et al.*, 2018). Unfortunately, the majority of these randomized controlled trials did not have infant growth as the primary outcome, but rather micronutrient homeostasis, such as iron and zinc. One randomized controlled trial in Germany (Dube *et al.*, 2010) compared two complementary feeding interventions with high (12%) and low (8%) meat content as a good source of dietary iron. Results showed no differences in terms of infant weight or length gain from 4 to 10 mo of age between groups. Although the sample size was relatively decent (*n* = 50 per group), the small differences in meat intake between groups (12% vs. 8%) could have contributed to the nonsignificant findings. Some other studies (Engelmann *et al.*, 1998; Makrides *et al.*, 1998; Krebs *et al.*, 2006) showing no effect of meat on infant growth also had short durations (~2 to 3 mo). One small randomized controlled trial (Tang and Krebs, 2014) found that compared with a plant/cereal based complementary diet (9% protein), a meat-based complementary diet (17% energy) promoted length gain from 6 to 9 mo in breastfed U.S. infants without increasing the risk of overweight (e.g., weight-for-length score). Another larger trial in China (Tang *et al.*, 2014) showed that meat consumption reduced stunting, namely, increased linear growth, in older infants. Several observational studies also looked at meat intake and the risk of overweight and obesity. Garden et al. (Garden *et al.*, 2011; Garden *et al.*, 2012) showed that the highest quintile of meat intake was significantly associated with greater BMI at 8- (Garden *et al.*, 2011) and 11-yr-old boys (Garden *et al.*, 2012). The same studies (Garden *et al.*, 2011; Garden *et al.*, 2012) also found that dairy products (combined milk and milk products such as yogurt and cheese) at 18 mo were negatively associated with adiposity at 8 yr. Studies that compared various dietary patterns during complementary feeding were primarily observational and results are mixed. One population-based study showed that compared with an omnivorous diet of meat, dairy, and fish, infants consumed a macrobiotic diet of rice, pulses and vegetables had retarded growth, fat, and muscle wasting. A recently published systematic review that included all the above-referenced studies by the B-24 project committee concluded that there is insufficient evidence to determine a relation between meat, dairy intakes, or various dietary patterns and incidence of overweight or obesity in older infants (English *et al.*, 2019).

Research in adults and animal models showed that types of protein-rich foods differentially affect various health indicators, including insulin resistance, cancer risk, and bone health. When considering the potential differential effects of types of protein-rich foods on infant growth, findings are extremely limited. One observational study from Germany using diet records showed that dairy intake at 12 mo of age was associated with body mass index at 7 yr, whereas meat, plant, or cereal was not, after controlling for confounders (Gunther et al., 2007). To determine the impact of types of protein on infant growth and risk of overweight, randomized controlled trials are preferred with growth parameters as the primary outcome and evaluates direct comparison of different types of protein-rich foods. One recently published randomized controlled trial compared two types of protein: meat and dairy during early complementary feeding on U.S. infant growth (Tang *et al.*, 2018). In brief, formula-fed infants from the metro Denver area were randomized to a meat- (*n* = 32) or dairy-based (n = 32) complementary diet from 5 to 12 mo of age. Total protein intake during the intervention was considered high at 15% of energy (~3 g/kg/d). The meat group consumed pureed beef, pork, and poultry (provided) and the dairy group consumed yogurt and cheese (provided). The same infant formula was provided for both treatments and fruits and vegetable consumptions were not restricted. Both groups consumed comparable amounts of total protein and minimum protein from the assigned (Tang *et al.*, 2018). Intakes of energy, formula, fruits, and vegetables also did not differ during the intervention. Results showed that that the length-for-age *Z* score (LAZ, the parameter of linear growth) increased in the meat group and decreased in the dairy group over the course of the intervention, which resulted in half-inch (2.5% or 1.8 cm) longer length in the meat group at 12 mo (Tang *et al.*, 2018). Because both groups gained comparable amounts of weight, this deceleration of linear growth in the dairy group led to a significant increase of weight-for-length score of 0.76 from 5 to 12 mo, which was considered accelerated gain and increased risk of overweight. Importantly, these growth patterns from 5 to 12 mo also persisted at 24 mo, 1 yr after the dietary intervention ended (Tang *et al.*, 2019). Specifically, the difference in length between meat and dairy groups remained at 1.9 cm at 24 mo. Participants from both groups consumed comparable amounts of protein from a mixture of meat, dairy, and plant. These findings suggest the long-term impact of early complementary feeding on growth and risk of overweight (Tang *et al.*, 2019).

Overall, the current literature provides insufficient evidence to advise dietary recommendations during complementary feeding, especially regarding protein-rich foods, on infant growth, and the risk of overweight. A limited number of randomized controlled trials showed that meat may be beneficial in terms of promoting length gain in infants, which subsequently reduced the risk of overweight. However, available randomized controlled trials of complementary feeding primarily focused on outcomes other than growth, and findings on body composition (% body fat) are scarce.

## The Early Protein Hypothesis

**Figure 2.**
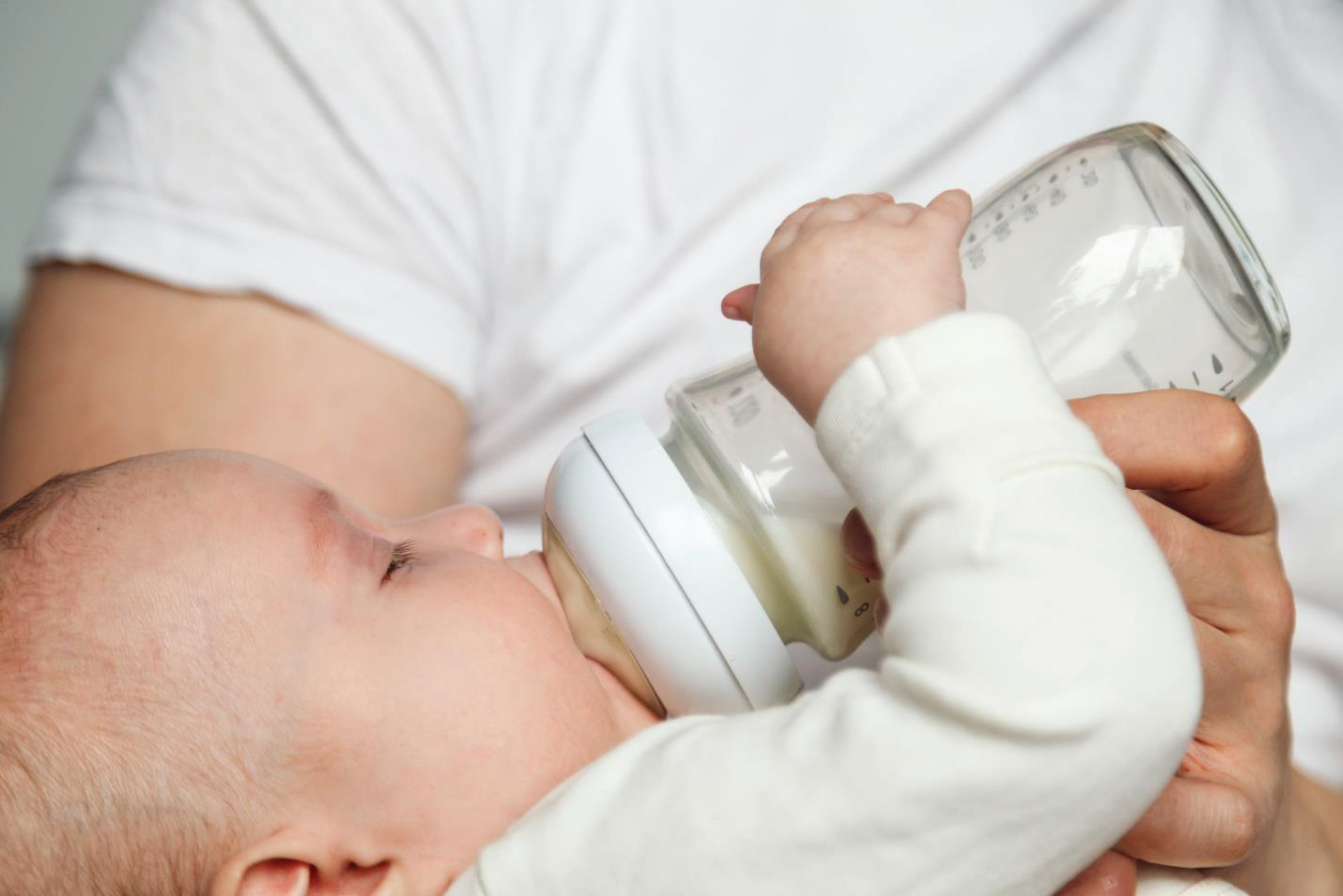
The Early Protein Hypothesis suggests that high-protein intakes accelerate weight gain in formula-fed infants.

The mechanisms linking protein intake and infant growth remain unclear. The Early Protein Hypothesis explained the observations that high-protein intakes accelerate weight gain in formula-fed infants. This hypothesis proposed that high protein intake may increase the secretion of insulin and insulin-like growth factor 1 (IGF-1) and result in rapid weight and fat gain in infants. The European Childhood Obesity Project tested this hypothesis in their multicountry randomized controlled trial using high- vs. low-protein infant formula (Koletzko *et al.*, 2009) and showed that IGF-I concentration was 40% higher in the high-protein formula group compared with the lower-protein formula group at 6 mo of age (Figure 2). Weight-for-length at 6, 12, and 24 mo was also positively associated with IGF-1 at 6 mo (Socha *et al.*, 2011). However, in the randomized controlled trial that compared meat and dairy proteins in formula-fed U.S. infants, IGF-1 significantly increased in both Meat and Dairy groups from 5 to 12 mo without group differences (Tang *et al.*, 2018), which was expected because both groups had a similar increased intake of protein quantity (2 to 3 g/kg/d). However, the increase of IGF-1 cannot explain the decline of linear growth (LAZ). Moreover, IGF-1 is one of the most copious growth factors in human bone and both animal and human studies showed that IGF-1 is associated with bone growth. Thus, the early protein hypothesis of IGF-1 inducing excessive weight gain relative to length gain is not supported by the literature and needs further justification. Emerging animal research suggests that the gut microbiome could play an important role in regulating growth trajectories.

## Effects of Complementary Foods on the Gut Microbiome

The role of the gut microbiome in human health and disease has been extensively examined, especially concerning body weight. A lower Bacteroidetes to Firmicutes ratio has been demonstrated as an “obesogenic” gut microbial profile and colonization of germ-free mice with “obesogenic” gut microbiota resulted in a significantly greater body fat increase than colonization with a “lean” microbiota. In preschool children, the dominating bacterial community tended to be less diverse in the obese/overweight children compared with those with normal BMI. Overall, the gut microbiota appears to have a significant impact on body weight regulation, as demonstrated in both human and animal studies.

**Figure 3.**
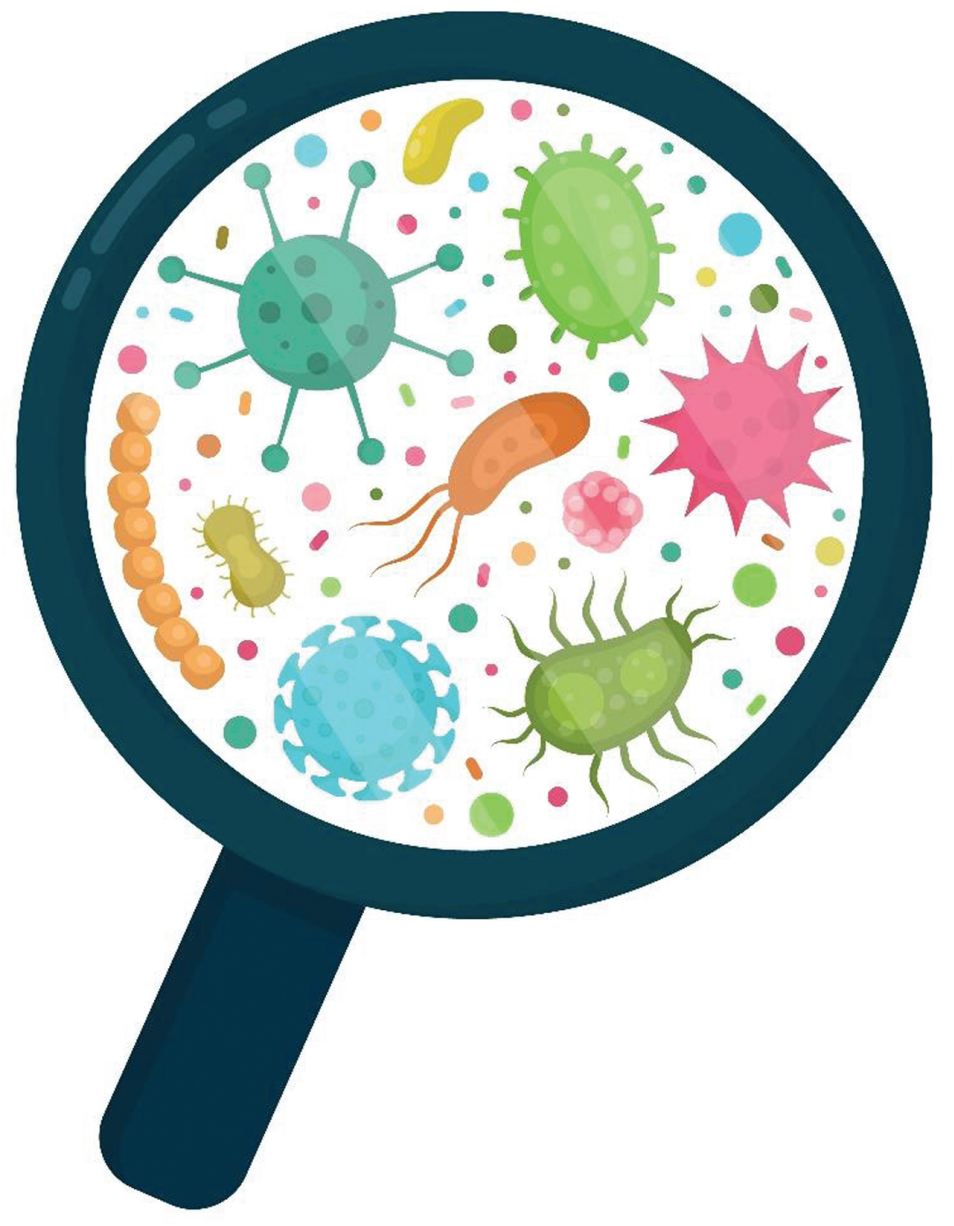
The infant’s gut is quickly colonized depending on feeding mode: breastmilk, infant formula, or both beginning at birth. Emerging research suggests that gut microbiota may affect infant growth.

Infancy is a life stage when the gut microbiota has low stability and high responsiveness to influencing factors, such as diet. Diet has significant implications for gut microbiota colonization (Laursen *et al.*, 2016). Besides maternal impact, the infant’s gut is quickly colonized depending on feeding mode: breastmilk, infant formula, or both beginning at birth (Figure 3). Among breastfed infants, *bifidobacteria* can account for over 60% of the total fecal microbiota, while formula-fed infants tend to have more *Bacteroides* and *Clostridium* (Collado *et al.*, 2012). Early complementary feeding is also a critical period of shaping the gut microbiome. After the “single food” phase of breastmilk or formula, complementary feeding is an important transition point that drives the pattern of maturation towards “adult” gut microbiome (Charbonneau *et al.*, 2016). However, despite comprehensive research on breastfeeding and the gut microbiome, very few studies have addressed the impact of complementary foods on the infant gut microbiota development. Magne et al. (Magne *et al.*, 2006) followed 11 infants longitudinally. *Ruminococcus* sp. was detected in one infant out of 11 during exclusive breastfeeding, and this number increased to 6 during weaning and postweaning period, suggesting the introduction of solid foods can change the gut microbiota. Another more recent cohort study (Laursen *et al.*, 2016) compared infants of both normal weight and obese mothers and found that protein and fiber intakes during early complementary feeding (9 mo of age) were associated with gut microbiota richness (alpha diversity). This study also found that protein intake was positively associated with increased abundance of *Roseburia* and *Ruminococcus*, independent of maternal BMI, delivery mode, or gestational age (Laursen *et al.*, 2016). Both *Ruminococcus* and *Roseburia* are considered key producers of extracellular glycosidases that degrade polysaccharides and produce short-chain fatty acids (Magne *et al.*, 2006). There are many health benefits of short-chain fatty acids that are well documented (Byrne *et al.*, 2015). Besides the beneficial impact on colon health, short-chain fatty acids are reported to activate G-protein coupled receptors (GRP41 and 43), which regulate fat accumulation and energy expenditure of the host (Kimura *et al.*, 2014). Emerging research also showed that short-chain fatty acids directly regulate bone metabolism in mice and prevents inflammation-induced bone loss (Lucas *et al.*, 2018). Overall, complementary feeding presents new energy and nutrient sources for gut microbes during late infancy and could result in selective advantages for specific microbes to establish in the gut (Laursen *et al.*, 2016).

Effects of different types of protein-rich foods on the gut microbiome are still being explored. One animal study (Zhu *et al.*, 2015) compared proteins extracted from meat, dairy, and plant on the gut microbiota and found significant differences in gut microbial profiles between the three protein sources. At the family level, *Lactobacillaceae* and *Ruminococcaceae* were the characteristic bacteria in rats fed with red meat proteins. Another earlier study by our group (Krebs *et al.*, 2013) in 6- to 9-mo-old infants showed that compared with a low-protein, cereal-based complementary diet (9% energy from protein), a high-protein, meat-based diet (17% energy from protein) increased the abundance of butyrate-producing strain *Lachnospiraceae*. These studies, although limited, suggest that besides dietary fiber, protein from animal origins, such as meat, may also promote the growth of short-chain fatty acids producting bacteria.

Plausible speculation of the differential impact of protein sources on the gut microbiome could be due to differences in substrate exposure. Ten percent or more of the ingested protein can reach the colon, and this amount is at least partially dependent on protein quality. According to the WHO, the digestible indispensable amino acid score (DIAAS) is the best method for evaluating protein quality (higher DIAAS suggests greater digestibility). The DIAAS is 143 for dairy (e.g., whole milk powder) 111 for beef, and even lower for plants (e.g., 47 for barley and 10 for cereal). Different DIAAS between dairy, beef, and plants suggest that at the same protein quantity, different types of protein may result in different amounts of substrates available for the gut microbiota. Amino acid compositions for various protein sources also differ. For example, meat, dairy, and plant proteins have varying amounts of branched-chain amino acids, with dairy being the highest. Branched-chain amino acid supplementation in mice increased the abundance of *Bifidobacterium* (Yang *et al.*, 2016). Moreover, undigested proteins and amino acids in the colon may serve as an additional substrate for short-chain fatty acid production next to nondigestible carbohydrates (Rasmussen *et al.*, 1988). One study (Zhu *et al.*, 2016) compared meat, dairy, and plant-protein extracts on short-chain fatty acid production and showed plant-protein led to the highest short-chain fatty acid production in mice. These differences in protein quality and amino acid compositions may at least partially contribute to the differential impact of protein source on the gut microbiome. When studying protein-rich foods, other compounds in these whole foods may also contribute to the outcome measures (e.g., the gut microbiome). Future studies that employ –omic-based analysis on protein-rich foods could further decipher food components that drive the health benefits, at the molecular level.

## The Gut Microbiome and Infant Growth

Besides having a significant impact on body weight and obesity in adults, emerging research suggests that the gut microbiota may also affect infant growth. A cohort study (Forbes *et al.*, 2018) showed that exposures of breastmilk or formula differentially stimulated the gut microbiota changes, which are associated with the risk of overweight in Canadian infants. A landmark study (Blanton *et al.*, 2016) identified bacterial species whose proportional representation defines a healthy and mature gut microbiota during the first year of life in Malawian infants. Specifically, deviation from the normal gut microbial composition resulted in “immature” gut microbiota and growth impairment (Blanton *et al.*, 2016). Moreover, transplanting the gut microbiota from stunted infants to germ-free mice also transmitted impaired growth phenotypes, and adding back two growth-discriminatory species (*Ruminococcus gnavus, Clostridium symbiosum*) could ameliorate the growth impairment in mice (Blanton *et al.*, 2016). Another animal study (Schwarzer *et al.*, 2016) found that the gut microbiota interacts with the somatotropic hormone axis to drive growth during the juvenile period, and *Lactobacillus plantarum* promoted juvenile growth in a strain-dependent manner. Interestingly, one animal study (Zhu *et al.*, 2016) showed that intake of meat protein extracts substantially increased *Lactobacillus* abundance in rats, which may partially contribute to some previous findings of meat promoting linear growth in infants. Overall, these studies suggest that the gut microbiota may have a direct impact on infant growth and could be a potential mediator linking the complementary feeding to infant growth trajectories.

## Future Directions

**Figure 4.**
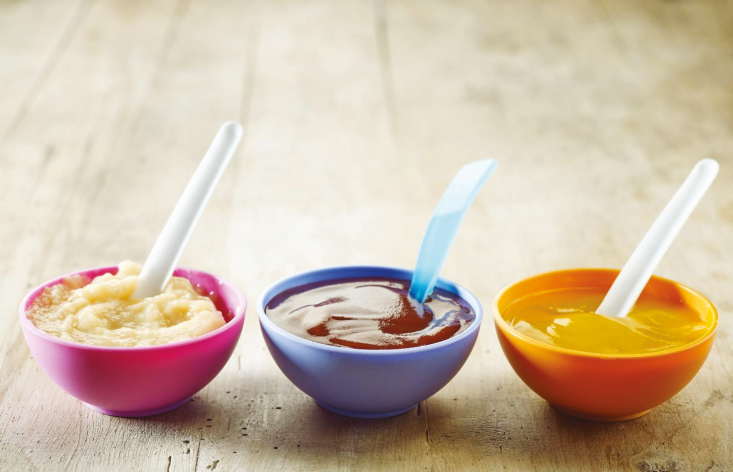
Early complementary feeding is a critical growth period that affects the etiology of obesity development. However, evidence-based dietary recommendations during early complementary feeding are lacking and more high-quality research is needed.

Early complementary feeding is a critical growth period that affects the etiology of obesity development. However, evidence-based dietary recommendations during early complementary feeding are lacking and more high-quality research is needed (Figure 4). Specifically, randomized controlled studies of dietary interventions with primary outcomes on infant growth and the risk of overweight are needed. Given the critical role of protein intake in infant growth and research priorites by the B-24 committee, dietary interventions on protein intake, both quantity and source, should be made a high priority. Infant outcomes also need to consider including not only growth *Z* score, but also body composition and neurodevelopment assessments. More importantly, mechanistic investigations of the impact of protein intake on infant growth are also lacking. Although imerging animal research showed a potential mediating effect of the gut microbiome in diet and growth, more human clinical studies that include longitudinal gut microbiome assessment are necessary.

## Acknowledgments

Funding was provided by NIH K01DK111665 to M.T.

## About the Author

**Dr. Minghua Tang** received her doctoral degree from Purdue University in 2012. She completed her postdoctoral training at the University of Colorado School of Medicine in 2016 and is currently an assistant professor at the University of Colorado. Dr. Tang’s research interests include the role of dietary protein in weight management and metabolic health, and both obesity treatment and prevention in adults and pediatric populations.
